# IL17RC affects the predisposition to thoracic ossification of the posterior longitudinal ligament

**DOI:** 10.1186/s13018-019-1253-3

**Published:** 2019-07-10

**Authors:** Peng Wang, Xiaoguang Liu, Xiao Liu, Chao Kong, Ze Teng, Yunlong Ma, Lei Yong, Chen Liang, Guanping He, Shibao Lu

**Affiliations:** 1 Department of Orthopedics, Xuanwu Hospital of Capital Medical University, 45 Changchun Street, Xicheng, Beijing, 100053 People’s Republic of China; 2 National Clinical Research Center for Geriatric Diseases, Beijing, 100053 People’s Republic of China; 3 Department of Orthopedics, Peking University Third Hospital, Beijing, 100191 People’s Republic of China; 4 Department of Radiology, Cancer Hospital Chinese Academy of Medical Sciences, Beijing, 100021 People’s Republic of China

**Keywords:** IL17RC, Thoracic ossification of the posterior longitudinal ligament

## Abstract

**Background:**

Thoracic ossification of the posterior longitudinal ligament (T-OPLL) can cause thoracic spinal stenosis, which results in intractable myelopathy and radiculopathy. The etiology of T-OPLL is unknown and the condition is difficult to treat surgically. Whole-genome sequencing identified a genetic variant at rs199772854 of the interleukin 17 receptor C (IL17RC) gene as a potentially pathogenic locus associated with T-OPLL. We aimed to determine whether the rs199772854A site mutation causes abnormal expression of the IL17RC in Han Chinese patients with T-OPLL and predict the possible pathogenic mechanisms of T-OPLL. Analyses were performed to determine whether IL17RC is involved in the pathogenicity of T-OPLL.

**Methods:**

Peripheral blood and OPLL tissue were collected from a total of 72 patients with T-OPLL disease (36 patients carrying the rs199772854A site mutation in IL17RC and 36 wild-type patients). The expression of IL17RC was analyzed by enzyme-linked immunosorbent assay, reverse transcription-quantitative polymerase chain reaction, immunohistochemistry, and Western blotting.

**Results:**

rs199772854A mutation resulted in markedly increased IL17RC gene expression levels in peripheral blood samples and the OPLL tissue obtained following clinical surgery (*P* < 0.05).

**Conclusions:**

The results suggest that the rs199772854A site mutation of IL17RC can significantly increase the expression of IL17RC. The IL17RC gene rs199772854A site polymorphism is a potential pathogenic mutation in T-OPLL disease, which may be associated with the occurrence of T-OPLL.

## Background

Thoracic ossification of the posterior longitudinal ligament (T-OPLL) is characterized by pathological heterotopic ossification of this region. T-OPLL is one of the common factors that cause thoracic spinal stenosis, which results in intractable myelopathy and radiculopathy. Early diagnosis of the disease is difficult, as the majority of patients develop symptoms only when the ossified ligament severely compresses the spinal cord. T-OPLL causes a much higher rate of disability than cervical OPLL (C-OPLL). T-OPLL has a marked ethnic predilection, as this disease occurs more frequently in Japanese and Chinese individuals. The prevalence of T-OPLL in individuals of Japanese ethnicity is 1.6–1.9%, and the mean age of onset is > 40 years old [1, 2].

Conservative treatments are typically ineffective for T-OPLL, with surgery as the only effective treatment. However, due to the unique anatomy and pathophysiological factors associated with for T-OPLL, postoperative cerebrospinal fluid leakage, paraplegia, infection, and other complications are common, occurring in 9.6–40.8% of patients that receive surgical treatment [3–5], and the pathogenesis of T-OPLL remains unclear. Most studies suggest that C-OPLL is a ‘genetic’ disease [6–16], the thoracic spine experiences less local biological stress than the cervical spine; thus, we speculate that genetic factors may contribute to the development of T-OPLL. Our previous whole-genome sequencing and candidate gene-association studies demonstrated that the presence of the rs199772854A single-nucleotide polymorphism (SNP) in the interleukin 17 receptor C (IL17RC) gene is potentially associated with T-OPLL susceptibility [17, 18]. Therefore, we hypothesize that IL17RC might be involved in the formation of OPLL of the thoracic spine.

The IL17RC gene is located at chromosome region 3p25.3-3p24.1 and encodes a type I transmembrane protein [19]. IL17RC is a proinflammatory receptor with a crucial role in the development of osteoblasts and accelerates osteoblast differentiation [20]. As OPLL promotes bone formation in ligament tissue, OPLL is associated with increased bone mineral density.

The present study aimed to determine whether the rs199772854A site mutation causes abnormal expression of the IL17RC gene in patients with T-OPLL among a Han Chinese population and to determine whether IL17RC is involved in the pathogenicity of T-OPLL.

## Materials and methods

### Disease criteria and patients

**Fig. 1:**
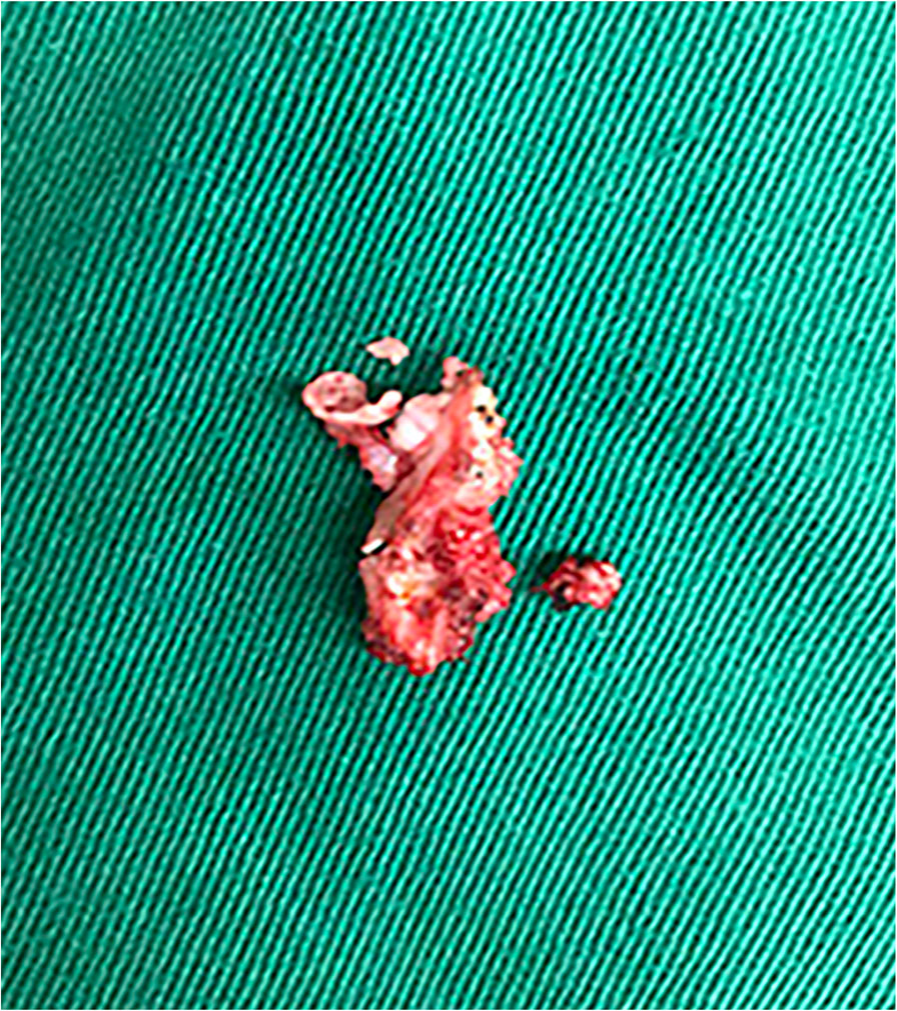
OPLL specimens of the thoracic spine. T-OPLL specimens removed by circumferential decompression surgery in patients with T-OPLL. *OPLL* ossified posterior longitudinal ligament, *T*-*OPLL* thoracic OPLL

This retrospective study protocol was approved by the ethical committee for human subjects of the Peking University Third Hospital (Beijing, China). Informed consent was provided by all participating individuals. Unrelated northern Chinese Han patients with T-OPLL carrying the rs199772854A site mutation in IL17RC and unrelated northern Chinese Han patients with T-OPLL carrying the wild-type rs199772854C site were enrolled in this study between May 2014 and July 2018. Diagnosis of T-OPLL was performed by orthopedic spine specialists based on clinical symptoms and computed tomography (CT) of the thoracic spine. The appearance of T-OPLL observed in CT was classified as segmental, continuous, mixed, or local disease type [21]. Neurological status was evaluated by the Japanese Orthopedic Association (JOA) score for thoracic myelopathy (maximum 11 points). The posterior longitudinal ligament specimens of the thoracic spine in patients with T-OPLL were collected during circumferential decompression surgery (Fig. 1).

### Plasma IL17RC enzyme-linked immunosorbent assay

Plasma collection and storage were performed using standard methods. Plasma IL17RC levels were quantified using commercially available enzyme-linked immunosorbent assay (ELISA) kits (Trust Specialty Zeal, Inc., San Francisco, CA, USA). All samples were assayed according to the manufacturer’s instructions and were run in duplicate. The optical density of each well was determined using a microplate reader at 450 nm. No interference and no cross reactivity were expected based on the manufacturer’s instructions.

### Reverse transcription-quantitative polymerase chain reaction

Total RNA was purified from blood using the SK1321 RNA Blood Mini Kit (Sangon Biotech Co., Ltd., Shanghai, China). A one-column DNase digest (Sangon Biotech Co., Ltd.) was performed before the clean-up step to eliminate residual genomic DNA. cDNA was synthesized from total RNA (2 μg) using a RevertAid Premium Reverse Transcriptase kit (Thermo Fisher Scientific, Inc., Waltham, MA, USA). Relative qPCR was applied to quantify the mRNAs levels of IL17RC using SYBR Green Real-Time PCR master mix on the LightCycler480 Real-Time System (Roche Diagnostics, Basel, Switzerland). All experiments were performed in triplicate and normalized to glyceraldehyde-3-phosphate dehydrogenase (GAPDH). Details of the primer sequences are listed in Table 1.

**Table 1:** Primer sequences used for quantitative polymerase chain reaction

Gene	Primer sequence
*IL17RC*	Forward 5′-TATGGGACGATGACTTGGGA-3′
	Reverse 5′-TGAGAAGGAGGATGAGGGAAA-3′
GAPDH	Forward 5′-TGGGTGTGAACCATGAGAAGT-3′
	Reverse 5′-GAGTCCTTCCACGATACCAA-3′

### Hematoxylin-eosin staining and immunohistochemistry analysis

Serial 5-mm-thick sections were prepared from paraffin-embedded thoracic spine specimens for staining. Hematoxylin-eosin staining was performed in an autostainer machine (Leica Microsystems GmbH, Mannheim, Germany) using standard procedures. Sections for immunohistochemical (IHC) staining were deparaffinized using xylene and dehydrated in serially graded ethanol solutions. The sections were washed in distilled water, treated with a 0.3% H_2_O_2_ solution dissolved in absolute methanol at 20 °C for 15 min, and then rinsed with PBS (pH 7.4). Antigen retrieval was performed using a high temperature and high pressure method. The sections were incubated with primary polyclonal mouse anti-human IL17RC antibody (1:200; ab69673; Abcam, Cambridge, MA, USA) at 4 °C overnight in a humidified chamber. Sections were washed with PBS three times for 5 min each wash and then incubated with horseradish peroxidase-conjugated goat anti-mouse IgG in a humidified chamber for 30 min at room temperature. Sections were rinsed with PBS (pH 7.4), and antibody binding was visualized by incubation with a diaminobenzidine (DAB) solution (ZLI-9017; OriGene Technologies, Inc., Beijing, China). Sections were washed in water to remove excess DAB and counterstained with hematoxylin to visualize nuclei. Negative control sections were incubated with PBS instead of the primary antibodies under the same conditions.

### Western blot analysis

Tissue lysates were obtained using ice-cold RIPA lysis buffer (Beyotime Institute of Biotechnology, Haimen, China) containing 100 mM PMSF as a protease inhibitor. Total protein (100 μg) was separated in a Bis-Tris polyacrylamide gel and transferred onto a nitrocellulose membrane. The membrane was then incubated in 1% bovine serum albumin containing primary rabbit anti-human polyclonal antibodies at 4 °C overnight. Following incubation with horseradish peroxidase-conjugated goat anti-rabbit antibody at room for 1 h, proteins were detected using electrochemiluminescence (EMD Millipore, Billerica, MA, USA). The following primary and secondary antibodies were used: anti-IL17RC (1:1,000; ab69673; Abcam) and goat anti-mouse antibody (1:2,500; CW0102M; Beijing Kangwei Century Biotechnology Co., Ltd., Beijing, China). The blots were detected using a Kodak film developer (Fujifilm, Tokyo, Japan). Protein levels were quantified by densitometry analysis using Image-Pro Plus 6.0 software (Media Cybernetics, Inc., Rockville, MD, USA). Beta-actin was used as the endogenous control.

### Statistical analysis

All statistical analyses were performed using SPSS v17.0 software (SPSS, Inc., Chicago, IL, USA). Descriptive data for continuous variables are presented as the mean ± standard deviations. Student’s *t* test was used to compare age, gender, and JOA score differences between T-OPLL patients with different IL17RC gene variants. The differences in T-OPLL subtypes between patients with or without IL17RC gene mutation were applied using one-way analysis of variance with post hoc Fisher’s test. *P* ≤ 0.05 was considered to be statistically significant.

## Results

### Genotype-phenotype analysis

A total of 36 unrelated northern Chinese Han patients with T-OPLL carrying the rs199772854A site mutation in IL17RC (16 men, mean age 56.11 ± 11.08 years; 20 women, mean age 53.25 ± 6.65 years) and 36 unrelated northern Chinese Han patients with T-OPLL carrying the wild-type rs199772854C site (14 men, mean age 58.43 ± 8.89 years; 22 women, mean age 53.45 ± 6.51 years) were enrolled in this study. Phenotype-genotype associations were analyzed among the T-OPLL patients with or without the rs199772854A mutation (*n* = 36 per group; Table 2). No differences were found between these two groups in terms of sex, age, and JOA score at diagnosis. Additionally, radiological analysis of T-OPLL morphology revealed that the mutation-positive patients and mutation-negative patients exhibited no difference in the disease type classification (segmental, continuous, mixed, or local).

**Table 2:** Clinical features of T-OPLL patients with or without rs199772854A mutation

Variable	rs199772854A (*n* = 36)	rs199772854C (*n* = 36)	*P*
Age (years)	54.61 ± 8.89	55.38 ± 7.79	NS
Male/Female	16/20	14/22	NS
Continuous	10 (27.8%)	14 (38.9%)	NS
Local	2 (5.6%)	2 (5.6%)	NS
Segmental	8 (22.2%)	10 (27.8%)	NS
Mixed	16 (44.4%)	10 (27.8%)	NS
JOA Score	3.29 ± 0.95	4.26 ± 0.45	NS

Data are presented as the means ± standard deviation or n (%). *T*-*OPLL* thoracic ossified posterior longitudinal ligament, *NS* not significant, *JOA score* Japanese Orthopedic Association scoring system for thoracic myelopathy (maximum 11 points)

### Analysis of IL17RC levels in the blood of patients with T-OPLL

**Fig. 2:**
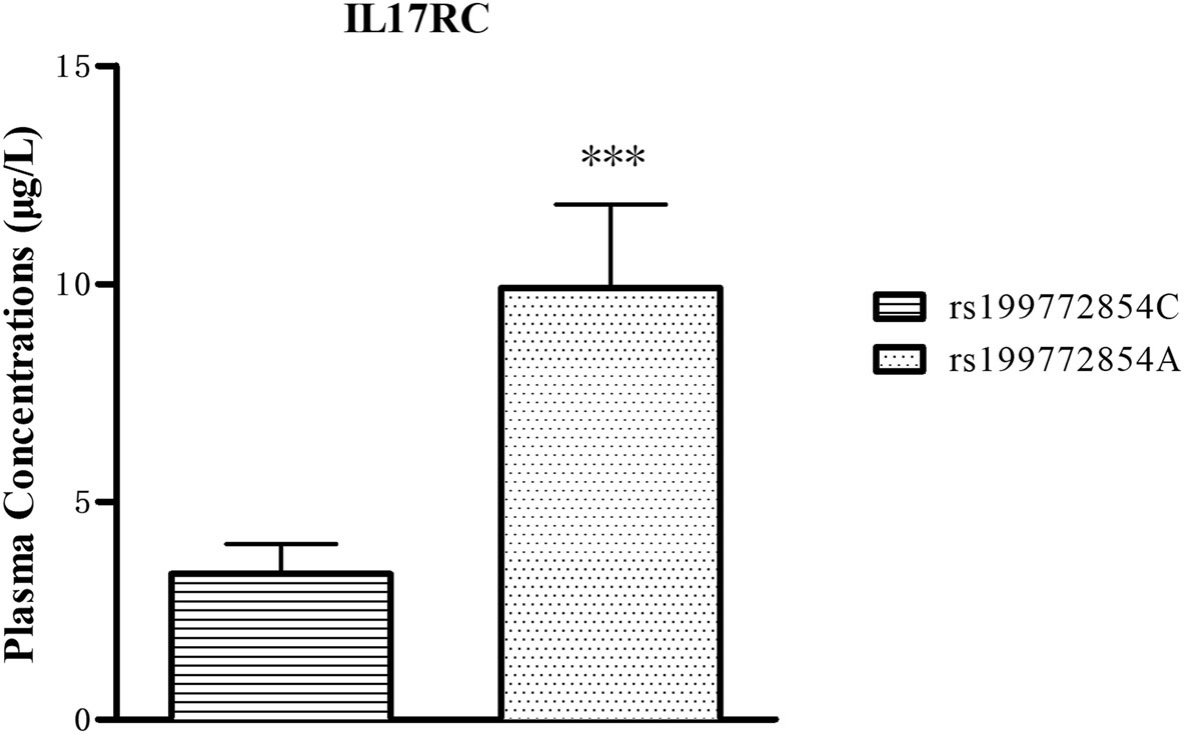
Plasma IL17RC ELISA. The plasma IL17RC level of T-OPLL patients with rs199772854A mutation was significantly higher than T-OPLL patients carrying the rs199772854C site. ***P < 0.001. *IL17RC* interleukin 17 receptor C, *T*-*OPLL* thoracic ossified posterior longitudinal ligament

**Fig. 3:**
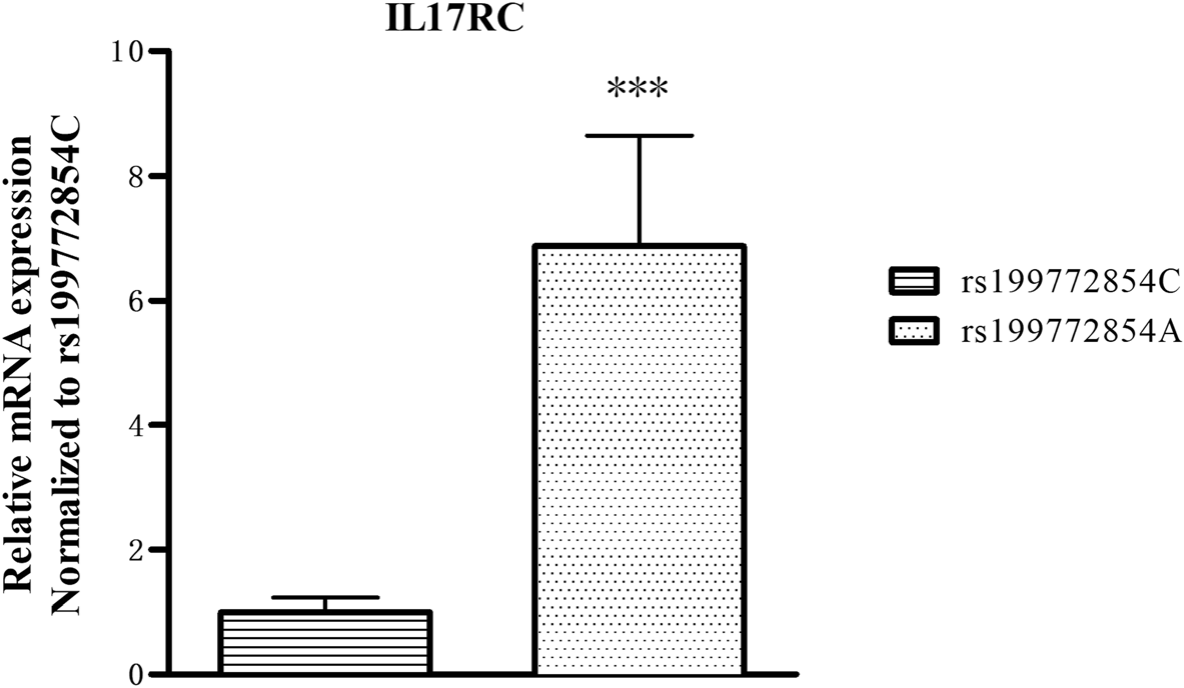
Analysis of IL17RC mRNA expression. The mRNA expression levels of IL17RC in T-OPLL patients with IL17RC gene rs199772854A mutation were significantly higher than that those T-OPLL patients carrying rs199772854C. ***P < 0.001. *IL17RC* interleukin 17 receptor C, *T*-*OPLL* thoracic ossified posterior longitudinal ligament

The plasma concentration of IL17RC was shown in Fig. 2; plasma IL17RC concentration was significantly higher (~ 3-fold higher) in T-OPLL patients with rs199772854A mutation (9.91 ± 1.91 μg/l) compared with T-OPLL patients carrying the wild-type rs199772854C variant (3.34 ± 0.69 μg/l; *P* < 0.01). Reverse transcription-quantitative polymerase chain reaction (RT-qPCR) analysis was performed using RNA extracted from peripheral blood cells (Fig. 3) and demonstrated that IL17RC mRNA levels were ~ 6-fold higher in T-OPLL patients carrying the rs199772854A mutation than T-OPLL patients with the wild-type rs199772854C site (*P* < 0.001). Compared with wild-type T-OPLL patients, the rs199772854A mutation significantly increased IL17RC gene expression, suggesting that this is a potential pathogenic locus that alters IL17RC gene expression in cells.

### IL17RC protein expression in T-OPLL patients with rs199772854A mutation

**Fig. 4:**
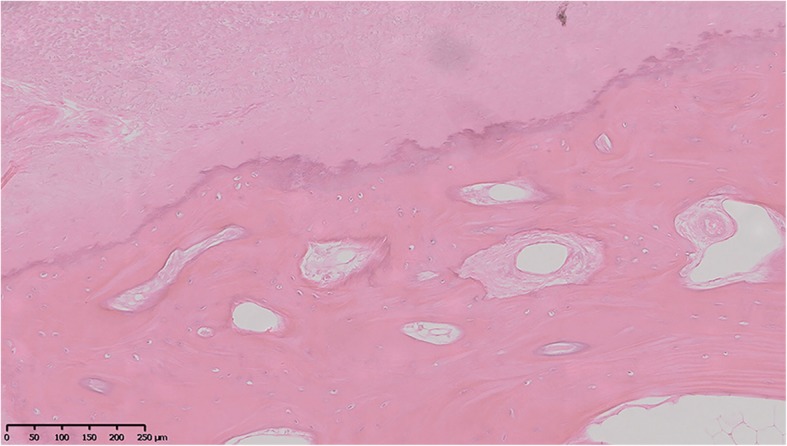
Hematoxylin-eosin staining. Representative hematoxylin-eosin staining of thoracic ossified posterior longitudinal ligament. Scale bar, 250 mm

**Fig. 5:**
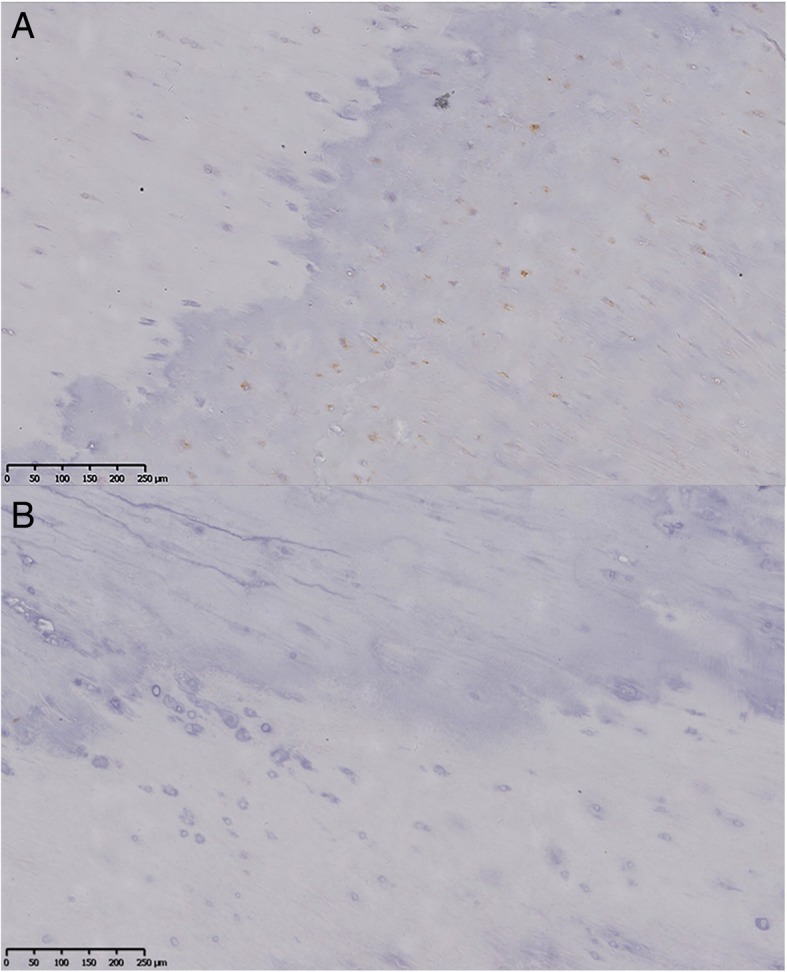
IHC staining. Representative IHC staining for IL17RC. **a** T-OPLL patients with IL17RC gene rs199772854A mutation. **b** T-OPLL patients carrying rs199772854C. Scale bar, 200 mm. *IHC* immunohistochemistry, *IL17RC* interleukin 17 receptor C

**Fig. 6:**
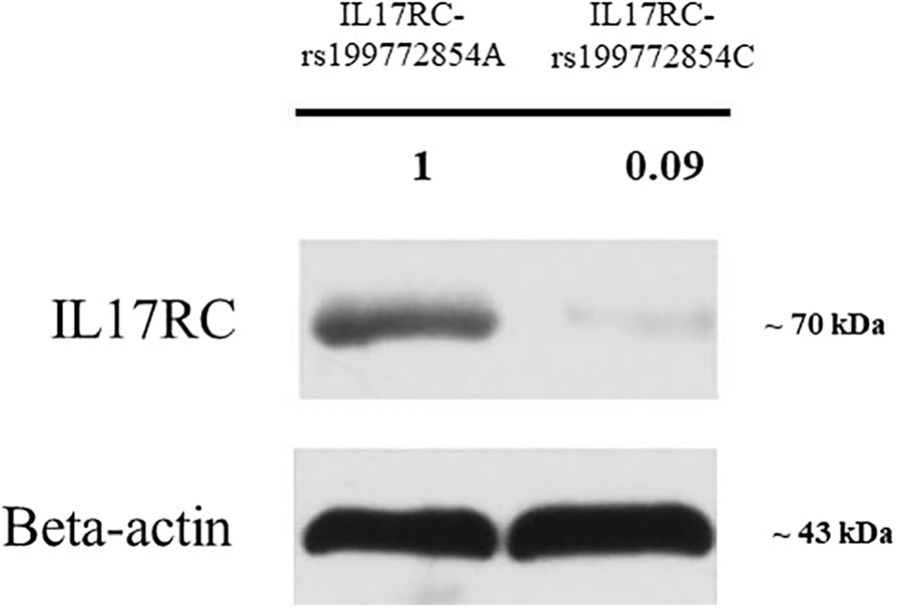
Protein expression of IL17RC. The expression levels of IL17RC protein in T-OPLL patients with IL17RC gene rs199772854A mutation was significantly higher than T-OPLL patients carrying the rs199772854C variant. *IL17RC* interleukin 17 receptor C, *T*-*OPLL* thoracic ossified posterior longitudinal ligament

Hematoxylin-eosin staining, shown in Fig. 4, revealed that there were more chondrocytes in the ossification zone (open arrows) and zigzag tidal traces formed by calcification were visible near the chondrocytes (solid arrows) in T-OPLL patients. IHC revealed that IL17RC protein was positively expressed in the ossified areas of T-OPLL patients with rs199772854A mutation, but no nuclear reactivity was observed in the normal fibers. However, there was weakly positive IL17RC in T-OPLL patients carrying the wild-type rs199772854C variant (Fig. 5). Western blot (WB) analysis revealed that the expression of IL17RC protein was significantly higher in T-OPLL patients with the IL17RC gene rs199772854A mutation than T-OPLL patients carrying the wild-type rs199772854C variant (Fig. 6).

## Discussion

T-OPLL poses a major challenge for spinal surgeons. Although the exact cause of the disease is still unclear, it is generally believed that genetic factors have an important role in the development of the disease. Some scholars believe that the accumulation of harmful missense mutations in the human genome creates the genetic basis for various complex diseases [22]. Whole genome sequencing identified a C > A mutation at the rs199772854 locus of the IL17RC gene. Disease progression is also affected by the gene expression in peripheral blood cells, with several genes reported to exhibit higher expression in the peripheral blood of OPLL patients compared to healthy controls [9, 23].

In this study, peripheral blood of T-OPLL patients with or without an rs199772854 mutation was collected and analyzed to assess the role of the mutated gene locus. The results of ELISA and qPCR analysis demonstrated that the expression of IL17RC was significantly higher in the peripheral blood of T-OPLL patients carrying the rs199772854A mutation compared to patients without the mutation. IHC and WB were performed to determine IL17RC expression in patients with T-OPLL, and the results demonstrated that the expression of IL17RC protein was significantly higher in the T-OPLL patients with the mutation compared to those with the wild-type. Therefore, the rs199772854A site mutation may lead to overexpression of IL17RC. We also showed that there was no difference in the OPLL classification and JOA score between T-OPLL patients with and without the rs199772854A mutation. The possible association between the rs199772854A site mutation in IL17RC and the severity of the T-OPLL phenotypes requires larger-scale studies in the future.

The rs199772854 site of the IL17RC gene is located in the promoter region. An SNP in the promoter region can increase or reduce gene transcriptional activity by altering the binding efficiency of transcription factors to various sequence elements, thus interfering with the gene expression process and potentially leading to disease occurrence. However, the mechanism and role of IL17RC gene regulation during ectopic osteogenesis remains unclear. IL17RC may accelerate bone metabolism via the transforming growth factor-β signaling pathway [24, 25]. The majority of studies suggest that IL17RC has a major role in disease pathogenesis through its function in the IL17 signaling axis. Some studies have shown that the IL17 axis affects bone formation and remodeling, and can also protect bone mass if bone loss occurs due to infection or hormonal imbalance [26, 27]. In addition, the IL17 axis can induce osteoblastic differentiation of bone marrow-derived mesenchymal stem cells [28]. It has become increasingly clear that inflammation-mediated imbalance in bone is a major feature of various bone diseases [29]; this imbalance is caused by increases in various cytokines in the inflammatory tissue. As an inflammatory factor, IL17 RC are expressed on various cells, such as osteoblasts, chondrocytes, and fibroblasts [30], and compression force can induces the expression of IL-17RC in osteoblast-like cells [31, 32]. In addition, the differentiation of osteoclast precursors into osteoclasts is suppressed by IL17RC [30]; IL17RC produced in response to compressive force may suppress osteoclastogenesis through the expression of OPG [31]. Additionally, IL17RC stimulates the secretion of other factors such as IL-6, tumor necrosis factor (TNF)-α and IL-1β in osteoclasts, further aggravating the inflammation [33].

The results of this study demonstrated that the expression of IL17RC in T-OPLL patients carrying an rs199772854A mutation was significantly higher in peripheral blood and tissues than in patients without the mutation. It is suggested that the rs199772854A site mutation can lead to overexpression of IL17RC and may induce pathological OPLL. However, this study lacks in-depth research and discussion on the mechanism by which IL17RC facilitates T-OPLL. In addition, due to the prevalence of T-OPLL disease is very rare, this study’s sample size is small. The association between heterotopic ossification and IL17RC requires further research.

## Conclusions

In conclusion, the findings of this study suggest that the rs199772854A site mutation can lead to overexpression of IL17RC and is a potential pathogenic mutation associated with T-OPLL. The results provide a potential basis for the pathogenesis of T-OPLL and the pathogenic role of IL17RC in T-OPLL disease.

Further genetic studies with more participants are required to validate these findings, and in appropriate model systems.

## Abbreviations

C-OPLL: Cervical ossification of the posterior longitudinal ligament
CT: Computed tomography
ELISA: Enzyme-linked immunosorbent assay
IHC: Immunohistochemical
IL17RC: Interleukin 17 receptor C
JOA: Japanese Orthopedic Association
RT-qPCR: Reverse transcription-quantitative polymerase chain reaction
T-OPLL: Thoracic ossification of the posterior longitudinal ligament
WB: Western blot
